# On the Nature of Scientific Progress: Anarchistic Theory Says “Anything Goes”—But I Don't Think So

**DOI:** 10.1371/journal.pbio.1001165

**Published:** 2011-10-04

**Authors:** Axel Meyer

**Affiliations:** Lehrstuhl für Zoologie und Evolutionsbiologie, Department of Biology, University of Konstanz, Konstanz, Germany

## Abstract

Evolutionary biologist Axel Meyer reviews the new English translation of philosopher Paul Feyerabend's *The Tyranny of Science*.

**Figure:**
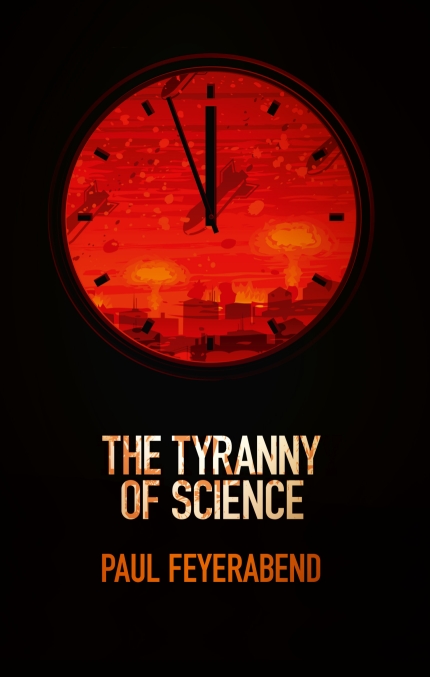
Feyerabend P (2011) The Tyranny of Science. Oberheim E, editor. Cambridge: Polity Press. 180 p. ISBN-13: 978-0745651897 (hardcover). US$54.95

Philosophy is the oldest of the disciplines that have been taught in places that we have called universities for centuries. Many of the readers of this journal have a PhD—nominally we are doctors of philosophy—even if you hadn't especially cared or even noticed. This is, of course, a hold-over of a history and tradition that dates back to the ancient Greeks and is based on the primacy of philosophy over all other academic pursuits.

But most scientists never even took a class in the history much less the philosophy of science. I would submit that only a small subset of practicing scientists might have actually stopped doing science and asked themselves questions such as: How does science work? How does it progress? Is there even progress in science? How is knowledge gained and accumulated? Scientists do strongly believe that there is progress, but you might be surprised that philosophers and other scholars in the humanities don't necessarily think so. Scientists nowadays do not tend ask themselves philosophical questions about the nature of science; they are too busy, they are preoccupied with figuring out how to get their papers published in journals such as *PLoS Biology* or how they will get their next grant application funded. Rarely, if ever, do they take the time to read what historians of science and much less philosophers of science think that they, the supposed study objects, actually do in their daily lives. If pressed, some researchers would, following Karl Popper's dictum, claim to be doing experiments in an effort to *falsify* a hypothesis, and to be working using the “hypothetico-deductive method.” But in unguarded moments they would say that they are collecting evidence “for” rather than “against” their favored hypothesis.

Self-reflecting scientists are surely going to encounter at least a small handful of philosophers of science during their ponderings. Karl Popper's *The Logic of Scientific Discovery* [1] and his falsification of hypotheses is probably on the top of the list. Next might be Thomas Kuhn, who in *The Structure of Scientific Revolutions* [2] developed the still prominent idea that a paradigm-driven phase of “normal science” may encounter anomalies that then can cause a crisis and eventually a scientific revolution and paradigm shift would be expected to follow. A very different view on how science advances was espoused by Paul Feyerabend (1924–1994) whose latest—posthumously published—book *The Tyranny of Science* [3] is the focus of this review. He is considered by many to be the third greatest 20th century philosopher of science. In his international bestseller from 1975 *Against Method* [4], Feyerabend said, “The only principle that does not inhibit progress is: anything goes” (p. 23) and “Unanimity of opinion may be fitting for a church, for the frightened or greedy victims of some (ancient, or modern) myth, or for the weak and willing followers of some tyrant. Variety of opinion is necessary for objective knowledge. And a method that encourages variety is also the only method that is comparable with a humanitarian outlook” (p. 46). Feyerabend argues strongly against the power that he sees science has: “The separation of state and church must be complemented by the separation of state and science, that most recent, most aggressive, and most dogmatic religious institution” (p. 295).

Before I go on I have to come clean on a couple of things. I have to admit that I hold a few prejudices against philosophers and even have a rather polemical relationship towards philosophy. This might prevent you from reading on. And this attitude will surely disqualify me with people in the humanities, but those people don't read science journals anyhow, apparently even some of those that philosophically interpret science for a living. In my opinion this makes it hard to take them seriously. And I am not alone. Even highly regarded philosophers, such as the late Richard Rorty from Stanford, espoused the—particularly in his circles—provocative view that philosophy as the seeker of absolute truths long ago lost its authority. That's maybe why he chose to teach in the comparative literature rather than the philosophy department.

It seems fair to say that most scientists don't care about philosophy or religion—they don't need miracles and gods to make sense of the world. We are content with materialistic explanations, thank you very much. The scientific laws and rules that scientists discover suffice for them and guides (or restricts?) their view of the world. And this materialism hurts the philosophers' pride. In turn, the philosophers' irrelevance for at least the daily life of most scientists, and the influence that scientists rather than philosophers now have on modern life, makes a surprising number of philosophers apparently distain science and scientists' power. The philosophers' traditional hold on explaining the world is threatened or even superseded by scientific insights and technological and biomedical progress. Moreover, most scientists' ignorance of history and philosophy is unfathomable to philosophers, adding insult to injury. But what do they expect? Where does rationality reside if not in the sciences?

Well, is that really true? I am sure that the majority of scientists don't even ask themselves these kinds of questions, since (at least I presume) most of us firmly believe that science is a deeply rational endeavor and exercise. At the beginning of the 20^th^ century the nascent discipline of philosophy of science still also strongly held this opinion. But—and this might surprise scientists—in the 1960s this traditional view on the science's rationality began to be challenged. Today, a rational view on science, so I am told, is seen as old-fashioned and seems to be even a minority opinion in philosophy of science departments. This change in fashion was due mostly to the American Kuhn and, most importantly, to his then still friend the Austrian-born Feyerabend. Since his *Against Method* [4], science is seen by many philosophers not as a rational exercise, but as one that takes place in a historical, social, and political context. Those factors are seen to exert far more influence on what science is done and how its results are interpreted and implemented than methodological principles or rational scientific thought. The philosophical Dadaist Feyerabend is often credited with this change of attitudes of philosophers of science.

Feyerabend actually published more books posthumously than while he was alive. The year after his death his autobiography *Killing Time* [5]—its title in German is *Zeitverschwendung* (which means waste of time)—appeared. It made clear that he was a cynic and a provocative clown, who also suffered from severe depressions. At times he actually seemed to get scared of the influence he had gained himself and reversed his message repeatedly throughout his career. He seemed to feel that he had wasted his time and those of others that actually took him seriously—an honest Dadaist. Fifteen years after his death, the first volume of his *Naturphilosophie* [6] was published. He began work on the planned three volumes of *Naturphilosophie* already in the early 1970s. It was thought that he had not finished it until a manuscript was discovered in the archives of my university's library that holds Feyerabend's papers. Without getting bogged down in details in *Naturphilosophie* he attempts to cover everything from cave drawings to nuclear physics and quantum theory and how it changed how the world is viewed, a subject that was so important to him that he had planned to devote the last two volumes of *Naturphilosophie* to it.

Paul Feyerabend surely was an interesting character. I actually experienced him first hand in his lectures in Berkeley in the 1980s when I was studying there for my Ph.D. in Zoology. In those years, Feyerabend held professorships both at the ETH in Zurich and in Berkeley attesting to his cloud. Early in his life he had studied theatre, something that obviously stayed with him. He was the consummate dandy and showman. Feyerabend had a limp from a war injury, but because of it (I had just learned about Amotz Zahavi's handicap principle), or in spite of it, he had a throng of attractive female students that followed him around and even carried his brief case to the lecture hall. This kind of stardom of the intellectual kind is something that is not as prevalent in the sciences as it apparently is in the humanities. The lecture hall was always packed with eager students who even sat on the floor in front of the podium. He was very entertaining and fun to listen to, but in the end his main message only seemed to be that there is no one method of how science works and how it advances, if it advances at all. The one who screams the loudest will get heard seemed to be his credo and he surely made a ruckus and—maybe occasionally to his own chagrin—he did get heard. This method for academic success is in my estimation something that will work better in the humanities, where one does not deal with measurements, data, or testable theories and falsifiable hypothesis, but rather with words alone.

*The Tyranny of Science* [3] is the English translation from the original, published in 1996 in Italian. The book is based on tape recordings of four lectures with the misleading titles: “Conflict and Harmony,” “The Disunity of Science,” “The Abundance of Nature,” and “Dehumanizing Humans.” I at least could not quite tell why the chapters have the titles that they do, because, for example, the first chapter mostly deals with different aspects of Greek philosophy, Homer, and the meanings of Greek tragedies. If you are curious about what Socrates said and how Parmenides got it all wrong these lectures provide an entry point that is educational. All chapters are easy to read. They hold your interest because of a barrage of seemingly unconnected tidbits of information and, for me at least, because of a bewilderment that someone can see the world so differently from what I was brought up to believe.

Feyerabend challenges some of what he considers to be modern myths about science, including the myth that “science is successful.” From reading his book one gets the feeling that he really does not like science or at least deeply distrusts the whole scientific enterprise and what he calls scientific ideology. He does not see a need to spend times in laboratories and to know what scientist actually do in their daily lives. Feyerabend clearly states that he does not want to learn how science is done—that's in his opinion not important, to him it's just background noise to the major events in science. Stances such as those made Feyerabend a guru of, so-called, postmodern pluralism. To a scientist this sounds crazy; we like to think that facts should inform one's idea of science—but this is surely too simple a thought for those in the parallel universe that is philosophy.

So what, you ask, if anything, is the relevance of Feyerabend to biologists? To be perfectly blunt I do not see much of any. Many would say that biology has taken over the role of the lead science since the revolution of molecular biology from physics. But this is something Feyerabend pretty much ignores. He only quotes from Jacques Monod's *Chance and Necessity* [7], and he mentions the Berkeley biochemist Daniel Koshland (1920–2007) in a negative sense about the costs of the human genome project. That's about it. Fittingly, Ernst Mayr in *The Growth of Biological Thought* [8] mentions Feyerabend only in passing in one sentence together with Kuhn in regards to the issue of progress in science.

Still, *The Tyranny of Science* really makes for an entertaining and thought-provoking read—even if it did not change my outlook on science and I would imagine that it will not affect yours either. His firework of admittedly interesting thoughts and observations will probably only make you realize how separate the two cultures of the sciences and humanities have become, even in a subject matter such as philosophy of science where at least one group of scholars purportedly cares about what the other does. Philosophical Dadaism a la Feyerabend will not help you get your next paper published. When your next grant is rejected and you read the panel's report, it might console you to have learned that the world is not a rational place, and even science might not always be. It is probably true that the rationality of science is only an imaginary idealistic supposition. But, if you are honest with yourself, you will say that you already knew that.

**Editors' note:** Does the cultural divide between science and the humanities, first articulated by C. P. Snow over 50 years ago, still exist between biology and philosophy? In a mini experiment to find out, we asked a philosopher and biologist to review the recent English translation of *Tyranny of Science*, by 20th century philosopher Paul Feyerabend, perhaps best known for rejecting the claim that science is a singular discipline unified by common methods and concepts.

**About the Author**

Axel Meyer is a Professor of Zoology and Evolutionary Biology at the University of Konstanz in Germany. He is interested in the origin of biological diversity at all levels of biological organization. His main study objects are fishes, in particular cichlid fishes, and their modes of speciation, phylogenetic patterns of relationships, and the evolution of their genomes.
